# Minimal Influence of Extracellular DNA on Molecular Surveys of Marine Sedimentary Communities

**DOI:** 10.3389/fmicb.2018.02969

**Published:** 2018-12-04

**Authors:** Gustavo A. Ramírez, Steffen L. Jørgensen, Rui Zhao, Steven D’Hondt

**Affiliations:** ^1^Graduate School of Oceanography, University of Rhode Island, Narragansett, RI, United States; ^2^K.G. Jebsen Centre for Deep Sea Research, Department of Earth Science, University of Bergen, Bergen, Norway; ^3^K.G. Jebsen Centre for Deep Sea Research, Department of Biology, University of Bergen, Bergen, Norway

**Keywords:** *e*DNA, extracellular DNA, necromass, marine sediment analysis, microbial ecology, paleome

## Abstract

Extracellular DNA has been reported to comprise a large fraction of total DNA in near-seafloor sediment. However, the potential effect of extracellular DNA, arising from dead or moribund cells, on sequencing surveys is a critical concern that has largely not been addressed for marine sedimentary habitats. To address this concern, we interrogated freshly collected Arctic and Pacific sediment for extracellular 16S rRNA genes using the photoactive DNA-binding dye Propidium Monoazide. Significant differences between relative abundances of total (intracellular + extracellular) Bacterial 16S rRNA genes and relative abundances of intracellular Bacterial 16S rRNA genes are only detected in three of twelve shallow [10 cm below seafloor (cmbsf)] samples. Relative abundances of total Bacterial 16S rRNA genes are statistically indistinguishable from relative abundances of intracellular Bacterial 16S rRNA genes in all interrogated samples from depths greater than 10 cmbsf. 16S rRNA gene sequencing shows that even where significantly higher abundances of extracellular genes are detected, they have little or no influence on prokaryote community composition. Taxon-level analyses suggest that extracellular DNA, arising from *in situ* death, may be sourced from different organisms in sediment of different ages. However, the overall effect of extracellular genes on sequencing surveys of marine sedimentary prokaryotes is minimal.

## Introduction

Despite extreme energy limitation (D’Hondt et al., 2002; Jørgensen and Marshall, 2016), marine sediment contains a vast microbial community (Kallmeyer et al., 2012) that remains active over geologically long time-scales (D’Hondt, 2004; Parkes et al., 2005; Schippers et al., 2005). Exponential declines in prokaryotic cell counts and community richness occur with sediment depth and sediment age (Kallmeyer et al., 2012; Walsh et al., 2016), indicating that most taxa from surface sediment communities are poorly equipped for long-term subseafloor lifestyles and die within the first few hundred thousand years following sediment deposition. Prokaryotic moribundity and death ultimately compromise cellular membranes, exposing cytoplasmic contents to the extracellular environment (Nyström, 2001; Cangelosi and Meschke, 2014).

The presence of extracellular (*e*)DNA has been reported from shallow (<10 cm depth) deep-sea sediment (Dell’Anno and Danovaro, 2005). However, the explicit impact of *e*DNA on high-throughput sequencing (HTS) surveys of marine sedimentary life is generally unconstrained. *In situ* cell lysis results in *e*DNA production. *e*DNA may be a cryptic source of electron donors and nutrients (N, P) (Finkel and Kolter, 2001) and/or influence the local genetic landscape (Lorenz and Wackernagel, 1994; Thomas and Nielsen, 2005).

Standard DNA extraction from marine sediment (Ramírez et al., 2018) involves indiscriminate lysis of living, dormant and dead cells, resulting in the co-extraction of intracellular *i*DNA (DNA from intact cells) and extracellular *e*DNA (DNA external to cells plus DNA from cells with damaged membranes) (Webster et al., 2003). The extent to which total *t*DNA (*i*DNA + *e*DNA) extracts from marine sediment faithfully represent potentially active populations of intact cells relative to detrital molecular signals from dead cells remains largely unexplored (Torti et al., 2015). The untested degree of co-extraction of *e*DNA and *i*DNA (Levy-Booth et al., 2007) is a fundamental concern for microbiological studies of subseafloor sediment because detrital genetic information may skew ecological molecular surveys that target extant communities (Pietramellara et al., 2008).

We use Propidium Monoazide (PMA) to discriminate *i*DNA from *t*DNA. Propidium Monoazide is a photo-active DNA-binding dye that, upon light exposure, covalently binds the double helix, inhibiting strand separation during PCR (Nocker et al., 2006). Intact cell membranes are impermeable to PMA. Consequently, application of PMA to environmental samples “silences” *e*DNA via non-amplification, resulting in the exclusive amplification of *i*DNA (Nocker et al., 2007). Comparisons of PMA-treated samples to non-treated controls show that non-treated controls significantly overestimate microbial loads and diversity, due to the presence of amplifiable *e*DNA, in canal sediment (Nocker et al., 2010), soil (Carini et al., 2016), foodstuffs (Li and Chen, 2013), and spacecraft-assembly cleanrooms (Vaishampayan et al., 2013; Mahnert et al., 2015; Moissl-Eichinger et al., 2015). This method draws strength from its simple application immediately following sample recovery. This timing is critical because microbial viability and membrane integrity both depend on time since sample recovery, as well as sample-handling procedures (see section “Materials and Methods – PMA Treatment Rationale,” below).

In this study, we address the effect of *e*DNA on important ecological metrics of subseafloor sedimentary communities, specifically: (i) 16S rRNA gene abundance, a common proxy for cell abundance, and (ii) prokaryotic community structure and composition based on HTS. To do this, we compare PMA-treated and untreated samples from the same sedimentary horizons to assess the influence of *e*DNA on *t*DNA-based metrics of microbial communities in near-surface (<10 cmbsf) and subseafloor sediment (>10 cmbsf) from the Arctic and Pacific oceans.

## Materials and Methods

### Site Descriptions

Arctic cores were collected during the RV *G. O. Sars* expedition to the Arctic mid-ocean spreading ridge system in the Greenland Norwegian sea in July–August 2016. Pacific cores were collected by RV *Sally Ride* expedition SR1703 to the United States–Mexico Border Lands in February 2017. Arctic gravity cores were collected at the following sites: Arctic Site 4: Lat: 72 16.761N, Lon: 01 41.991E, water depth: 2,668 m and Arctic Site 5: Lat: 76 54.766N, Lon: 07 07.491E, water depth: 3,007 m. Pacific piston and gravity cores were obtained from the following sites: Pacific Site 1: Lat: 32 48.6971N, Lon: 119 56.5952W, water depth: 914 m; Pacific Site 2: Lat: 33 04.899N, Lon: 117 57.613W, water depth: 1,173 m; and Pacific Site 3: Lat: 33 00.950N, Lon: 117 57.613W, water depth: 945 m.

The Arctic sites differ significantly from the Pacific sites in sediment type and sedimentation rates. Comprehensive descriptions of nearby sites in the Pacific (ODP site 104) and Arctic (Northern Knipovich Ridge an Southern Mohns Ridge) are found elsewhere (Lyle et al., 1997; Hellevang and Pedersen, 2005). Sediment at the mid-Arctic ridge sites consists predominantly of hemipelagic and glaciomarine deposits. Sediment at the Pacific sites is siliciclastic clay, containing foraminifera and calcareous nannofossils.

### Geochemical Analyses

Briefly, oxygen measurements were performed using optodes and nitrate was measured using ion chromatography with UV-absorbance, using the techniques described by (D’Hondt et al., 2015). Ammonium concentrations were measured using the fluorometric technique (Holmes et al., 1999). Total organic carbon in sediment and porewater was measured using the pyrolysis procedure (Verardo et al., 1990).

### PMA Treatments

Sterile cut syringes were used for aseptically sampling split cores in the Arctic and sectioned core ends in the Pacific. For each sampled depth horizon, six 1.5 ml translucent microcentrifuge tubes each received 0.1 cm^3^ of fresh (never frozen) wet sediment, from syringe minicores. Three randomly selected tubes containing sediment had PMA added to a final concentration of 20 μM in 1× sterile phosphate buffer saline in a final volume of 1 ml (Moissl-Eichinger et al., 2015). The remaining three tubes containing sediment were treated as no-PMA controls and had only 1× phosphate buffer saline added to 1 ml volumes. A complete list of sample site/core/depth/treatment is found in the supplementary package (Supplementary Table S1).

Propidium Monoazide-treated samples and controls were incubated in darkness at 25°C and 1 atm for 1 h with slow mixing via hand inversion every 5 min. Subsequently, PMA-treated samples and no-PMA (control) samples were exposed on ice to a 500 W halogen lamp at a distance of 15 cm for 15 min with hand mixing via inversion every 5 min. Following light exposure, all samples were stored at -80°C and transported back to shore for DNA extraction, 16S rRNA gene quantification and sequencing.

### PMA Treatment Rationale

Propidium Monoazide efficiency completely relies on cell membrane integrity. Since some of our sediment samples are tens of thousands of years old, their communities’ cellular membranes may be particularly susceptible to damage. Consequently, our PMA protocol focuses on minimizing artifactual *e*DNA spikes by preventing inadvertent cell membrane damage. Toward this end, our protocol entails (i) working exclusively with freshly collected, never frozen, samples, (ii) gentle hand-mixing, rather than vortexing, during dark and light homogenization steps, and (iii) light exposure on ice to prevent thermal-induced membrane damage. In control experiments, our PMA-treatment protocol removed 73–98% of extracellular DNA depending on particle type (Supplementary Figure S1). We expand on these points as follows:

(i) Exclusive use of fresh, never frozen, samples: We did not freeze sediment samples prior to our extracellular DNA discrimination step. We exclusively used fresh samples and exposed them to PMA as quickly as possible because freezing, even with addition of a cryoprotectant, always damages a fraction (sometimes a large fraction) of the intact cellular community and causes cells to leak (Postgate and Hunter, 1961). Such damage will lead to artifactual spikes in *e*DNA content.
(ii) Gentle hand-mixing rather than continuous vortexing of sediment slurries: We gently hand mixed during dark incubation and light exposure. We did not use continuous vortexing (Carini et al., 2016) because we were concerned that beating of sediment particles against cells during vortexing mimics DNA extraction *via* “bead beating” or physical lysis (Miller et al., 1999). In this way, it may inadvertently disrupt intact cellular membranes and artificially inflate *e*DNA.
(iii) Propidium Monoazide photo-activation on ice: Industrial (500 W+) halogen lamps produce substantial amounts of heat. This issue is exacerbated by the short distance (∼15 cm) that is used for PMA-DNA covalent cross-linking (photo-activation). Consequently, thermally induced membrane damage is a potential concern for creating artifactual *e*DNA in the sample. To minimize this effect, we followed the recommendation of (Vaishampayan et al., 2013) by photo-activating the PMA on ice for all of our samples.

### DNA Extraction, Amplification, and Sequencing

DNA extractions were performed using the FastDNA Spin Kit for Soil (MP Biomedicals, Santa Ana, CA, United States) following the manufacture’s protocol. Sample blanks, with no sediment added, were extracted in parallel with sediment extractions for each extraction kit utilized. Extracts were cleaned with AMPure XP beads, following manufacture’s instructions, and quantified using Qubit^TM^ 2.0. The 16S rRNA gene V4 hypervariable region was targeted using the following universal prokaryotic primer set: 518F (5^′^-GTG YCA GCM GCC GCG GTA A-3^′^) and 806R (5^′^-GGA CTA CNV GGG TWT CTA AT-3^′^), with partial Nextera adapters (Caporaso et al., 2012). PCR amplification reactions were performed in triplicate for each extract using the following thermocycling program: 94°C for 3 min; 32× (94°C for 45 s, 50°C for 60 s, 72°C for 90s); 72°C for 10 min and a 4°C hold. Lastly, pooled triplicate amplicons were cleaned with AMPure XP beads. Sequencing of pooled triplicates was performed at the University of Rhode Island Next-Generation Sequencing (NGS) Facility using the Illumina MiSeq platform with V2 chemistry kit (2 × 250 bp, 500 cycles) reagents. All sequence data has been submitted to the NCBI SRA repository and may be accessed under BioProject PRJNA423269; BioSamples SAMN08223814: SAMN08223869.

### V4 Amplicon Sequence Processing and Analyses

Amplicon sequences were processed with *mothur* v.1.34.4 (Schloss et al., 2009) following the *mothur* Illumina MiSeq Standard Operating Procedure (Kozich et al., 2013). Briefly, forward and reverse reads were merged into a combined total of 7.61 million contigs. All paired reads with homopolymers longer than 6 base pairs, minimal length of 288 bp, maximum length of 294 bp, and any ambiguities were discarded. A total of 4.74 million contigs met these criteria. To ensure quality control and mitigate extraction-kit contamination (Salter et al., 2014), all sequences identified in sample blanks were removed from all other sample groups as recently suggested (Sheik et al., 2018), eliminating the blank extraction groups entirely from downstream analyses and paring the dataset to 3.46 million contigs. Reads were aligned to the *mothur*-recreated Silva SEED v119 database (Yarza et al., 2010), trimmed to the V4-hypervariable alignment region and subsequently pre-clustered at 1% dissimilarity using the *pre.cluster* (diffs = 3, for ∼300 bp amplicons, as suggested in the *mothur* SOP) command. Spurious sequence generation was mitigated by abundance-ranking sequences and merging with rare sequences if sequences differed by three base pairs (Kozich et al., 2013). Chimera screening and removal from further downstream analyses was performed by implementation of *de novo* mode of UCHIME (Edgar et al., 2011). Following the removal of chimeric sequences, the full Pacific and Arctic data set was randomly subsampled from 3.1 million to 400,000 contigs. A summary of group-specific sequence numbers after each quality control command is provided in the supplemental information (Supplementary Table S1). Using the average neighbor method, a distance matrix was generated, clustering sequences into operational taxonomic units (OTUs) at 3% or higher similarity cut off. Taxonomic classification of OTUs was performed with *mothur* using the SILVA v119 database (Quast et al., 2013). All analyses were performed on a rarefied dataset standardized to equal sample sizes (*n* = 3,591, per group, Supplementary Table S1). Individual per core clustering (3% similarity cut off) was also performed as described above and observed OTUs numbers were calculated with *n* = 28,481 sequences per group, for all Arctic and Pacific sites. Taxon-specific viability ratios (*i*DNA/*t*DNA), were computed for high-abundance OTUs. Taxa with *t*DNA abundance of zero are omitted, ratios larger than 1 are plotted as 1 (their theoretical maxima). Metrics for community richness, diversity and evenness, as well as Principal Coordinate Analysis, using Bray-Curtis distances, were performed in *RStudio* version 0.98.1091 (Racine, 2012) using the packages *vegan* version 2.3-0 (Oksanen et al., 2015) and *phyloseq* (McMurdie and Holmes, 2013). Community taxonomic heat maps were generated using the *metacoder* (Foster et al., 2017) R package.

### Quantitative PCR

For all DNA extracts that underwent 16S rRNA gene V4 sequencing, Bacterial 16S rRNA gene abundances were measured with Bacteria-specific primers Bac341f (5^′^-CCTACGGGWGGCWGCA-3^′^) and Uni518r (5^′^-ATTACCGCGGCTGG-3^′^) using the following thermocycling program: 95°C for 15 min, 40× (95°C for 15 s, 58°C for 30 s, 72°C for 30 s). Standards consisted of dilutions of size-verified, gel-extracted, purified PCR amplicon (*Escherichia coli* DNA, using the same primer set). A range of 10^2^ to 10^8^ 16S rRNA gene fragments per microliter for abundance standards was used in each run. The *R*^2^ value of all standard curves was >0.98 with estimated minimum amplification efficiencies of 110%. All samples, each representing experimental triplicates (see section “PMA Treatments” above), were run in technical triplicates (i.e., three qPCR values per experiment); thus *n* = 9 for each data point shown, standard error of the mean is smaller than the symbols. All quantitative PCR work was conducted at the Marine Science Research Facility at the University of Rhode Island on a Stratagene QPCR Mx3000P cycler.

## Results

### Porewater Geochemistry and Total Organic Carbon

**FIGURE 1:**
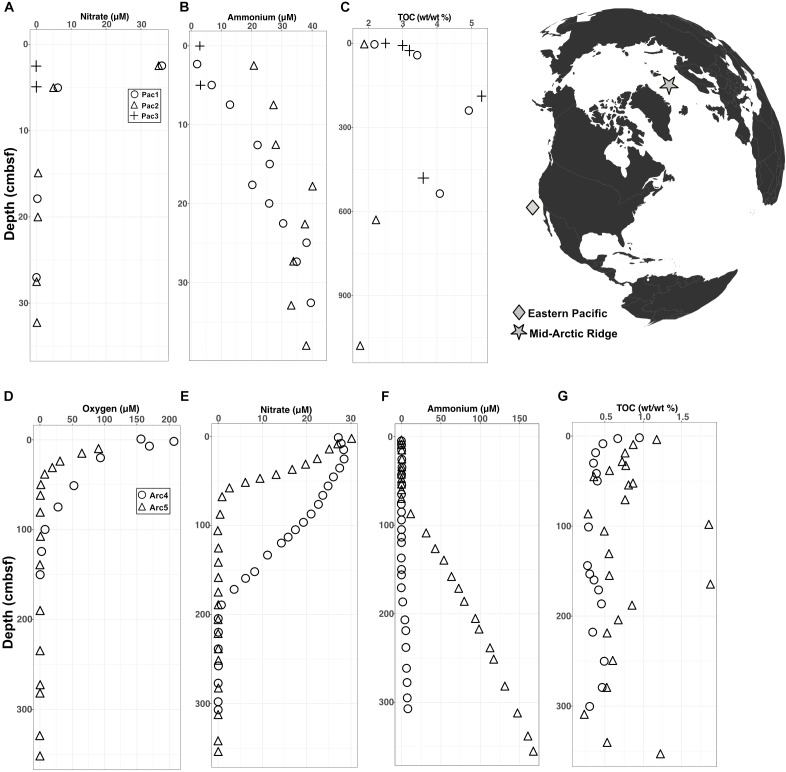
Geochemical data for Pacific and Arctic sites superimposed on a map of collection sites. **(A–C)** Depth variation of interstitial **(A)** nitrate, **(B)** ammonium, and **(C)** total organic carbon at the Pacific sites. Oxygen was below detection at all depths at all three Pacific sites. **(D–G)** Depth variation of interstitial **(D)** oxygen, **(E)** nitrate, **(F)** ammonium, and **(G)** total organic carbon at the Arctic sites.

The Arctic and Pacific sites differ drastically from each other in oxygen penetration depth and organic carbon content. In the Pacific cores, interstitial oxygen was below the detection limit at every depth, nitrate was depleted near the water-sediment interface, ammonium concentrations increased with depth, and total percent organic carbon (TOC) was high, ranging between 2–5% (Figures 1A–C). In the Arctic cores, oxygen levels were high at the water-sediment interface and penetrated down to ∼1 meter below seafloor (mbsf), nitrate depletion followed oxygen depletion, ammonium levels increased at depth, and TOC was low (largely < 1%) relative to TOC in the Pacific sites (Figures 1D–G).

### Quantitative PCR

**FIGURE 2:**
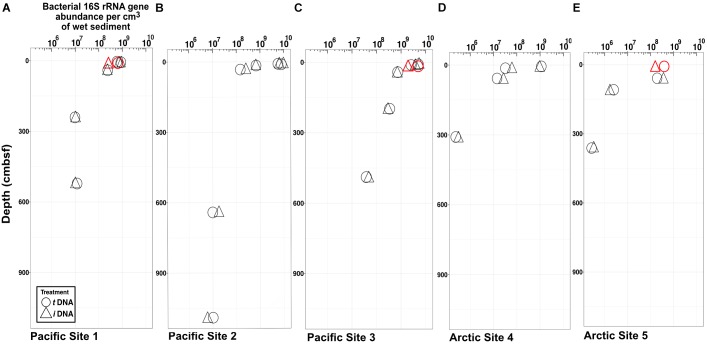
Bacteria-specific 16S rRNA gene q-PCR results for *total* DNA (circles) and *intracellular* DNA (triangles). Differences between relative abundances of total 16S rRNA genes (*t*DNA) and relative abundances of intracellular 16S rRNA genes (*i*DNA) represent extracellular DNA (*e*DNA). The symbols are larger in size than the standard error of the mean. Each sample pair (*i*DNA and *t*DNA) was tested for differences in reported relative gene counts (Student’s *t*-test). Three sample pairs exhibit significant differences (*P* < 0.05) and are depicted in red. **(A)** Pacific Site 1; **(B)** Pacific Site 2; **(C)** Pacific Site 3; **(D)** Arctic Site 4; and **(E)** Arctic Site 5.

Maxima and minima in abundance of bacterial 16S rRNA genes, respectively, occur in the shallowest and deepest horizons, with higher abundances in the Pacific (continental margin; Figures 2A–C) relative to the Arctic (open ocean; Figures 2D,E). For all depths in all sites, we compared gene abundances from PMA-treated samples and non-PMA-treated controls as proxies for abundances of intracellular *i*DNA and total (intracellular + extracellular) *t*DNA. Most horizons from both oceans show no statistically significant differences (Student’s *t*-test, α = 0.05) between PMA-treated (*i*DNA) samples and non-treated (*t*DNA) controls. In single near-seafloor samples from two Pacific sites and one Arctic site, non-PMA treated controls contain significantly higher (*P*_val_ < 0.05) 16S rRNA gene abundances than PMA-treated replicates (Figures 2A,C,E).

### The Effect of Extracellular DNA on 16S rRNA Gene Community Composition

**FIGURE 3:**
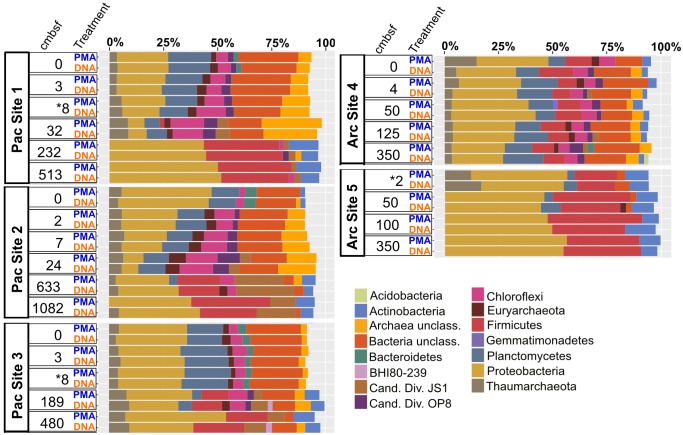
Phylum-level community composition for all sample pairs from all sampled depths in all Pacific and Arctic sites. Depth horizons where significant differences in relative abundances of intracellular 16S rRNA genes and total 16S rRNA genes were detected (see Figure 2) are depicted with asterisks.

**FIGURE 4:**
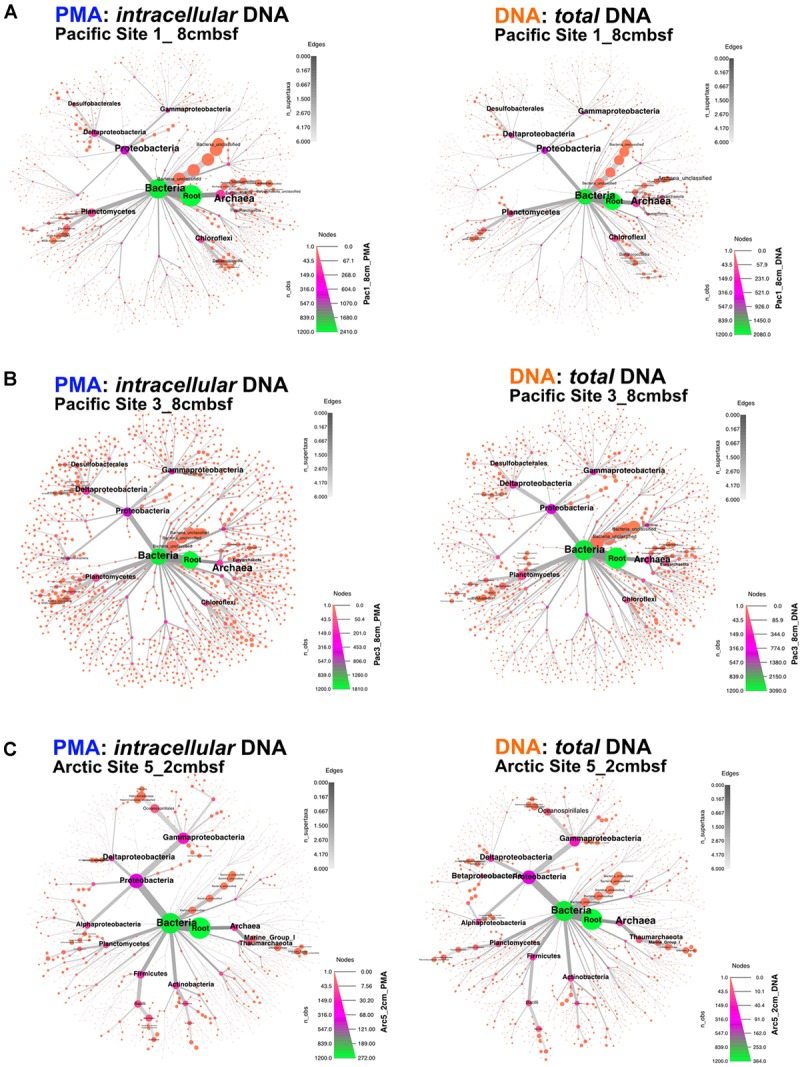
For the three sample pairs where statistically significant differences in *i*DNA and *t*DNA 16S rRNA gene quantities were found, hierarchical structure of taxonomic classification for *i*DNA (left) and *t*DNA (right) microbial communities is shown as heat trees. Size and color of nodes and edges represent the relative abundance of organisms from each community. **(A)** Pacific Site 1, 8 cmbsf; **(B)** Pacific Site 3, 8 cmbsf; and **(C)** Arctic Site 5, 2 cmbsf.

Archaea and Bacteria were detected in both Arctic and Pacific samples. At both sites, the relative abundance of Bacteria is higher than that of Archaea for all samples from all depths (Figure 3). Archaeal percent abundance is most pronounced in relatively shallow depths at each site and taxonomically resolved to Thaumarchaeota and Euryarchaeota, with varying contributions from unclassified Archaeal phyla. Proteobacteria, Planctomycetes, Firmicutes, Chloroflexi and candidate phyla OP8 (Aminicenantes), JS1 (Atribacteria) and BHI80-139 dominate the Bacterial sequences with phylum-level assignment (Figure 3). In Pacific sites, the relative contribution of unassigned Bacteria sequences diminishes with increasing depth and deeper communities become heavily dominated by the Proteobacteria, Firmicutes, Atribacteria and Actinobacteria nearly exclusively. In Arctic sites, phylum-level relative abundance changes less drastically with depth and Proteobacteria, Firmicutes, Atribacteria, and Actinobacteria remain dominant throughout. Phylum-level community compositions of PMA-treated samples are nearly identical to community compositions of non-treated samples for all horizons in both Pacific and Arctic sites. Even the paired PMA-treated and non-PMA-treated samples with significantly different 16S rRNA gene loads had nearly identical phylum-level taxonomic compositions (Figure 3, asterisks). For these three pairs with significant *e*DNA 16S rRNA gene abundances, the heat plots in Figure 4 illustrate the hierarchical taxonomic structures of communities as reported by *i*DNA and *t*DNA. This shows that community composition at multiple taxonomic hierarchies (class- and order-level), from *t*DNA highly resembles that of the *i*DNA (Figure 4).

### Ordination

**FIGURE 5:**
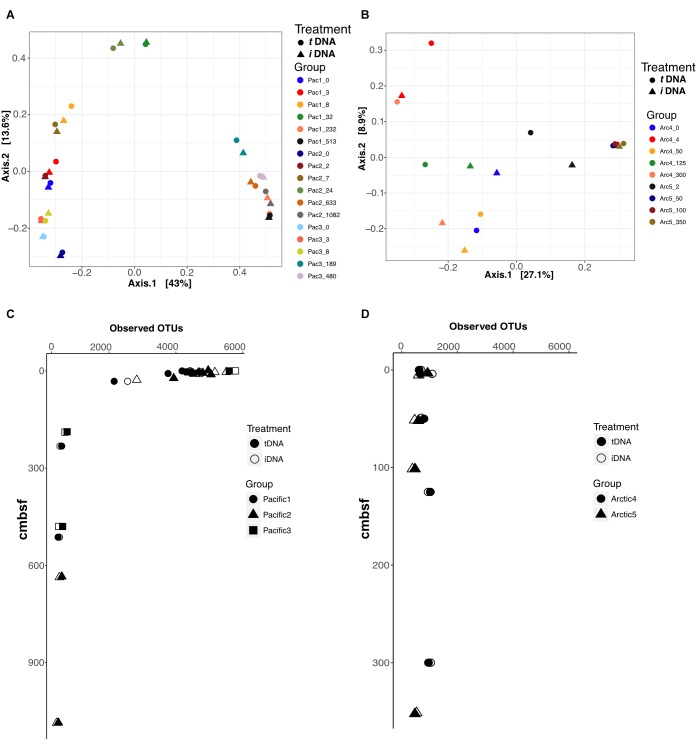
Principal Coordinate Analyses (PCoA) using Bray-Curtis distances to compare effects of *i*DNA and *t*DNA pools on 2-dimensional ordination for all sample pairs in **(A)** Pacific and **(B)** Arctic. Observed OTUs for all sample pairs in Pacific **(C)** and Arctic **(D)** data. Filled and open symbols represent *t*DNA and *i*DNA datasets, respectively.

We performed Principal Coordinate Analysis (PCoA) of Bray-Curtis dissimilarity distances for each sampling location using the 16S rRNA V4 sequence data, and mapped the results in 2-dimensional space (Figures 5A,B). For the Pacific, the first axis explains 43% of total variance and resolves shallow (axis 1 < 0.2), mid (-0.1<axis 1 < 0.1), and deep (axis 1 > 0.3) samples across all sites with close coupling of PMA treatments and non-PMA treated controls for all sample pairs (Figure 5A). For the Arctic, the first axis explains 27.1% of the total sample variance and bifurcates the plot by site, by assigning negative and positive axis 1 values to Site 4 and Site 5, respectively (Figure 5B). PMA treatment plays a role in the placement of Arctic sample pairs, particularly in Site 4. The largest differences based on PMA treatment are observed along axis 2, which only explains 8.9% of the variance of the total Arctic dataset. Conversely, Arctic Site 5 shows close coupling between PMA-treated and non-PMA-treated sample pairs from depths greater than 50 cmbsf (for which intergroup clustering is also observed) (Figure 4B). The ordination patterns are driven by Bacteria rather than Archaea in our data, as shown by exclusively plotting Bacteria data and observing identical patterns (Supplementary Figure S2).

### Observed OTU Counts

We calculated the number of observed OTUs for all samples in Pacific and Arctic sites (Figures 5C,D). At Pacific sites, number of OTUs declines with increasing sediment depth (Figure 5C). PMA-treated samples and untreated controls both show high diversity near the interface and low, nearly identical, diversity at depth. At Arctic sites, OTU numbers remain relatively steady with depth; as for the deeper Pacific horizons, the diversities of PMA-treated samples and untreated samples are nearly identical for each Arctic horizon (Figure 5D). At the shallowest site depths, taxonomic richness appears lower in the Arctic sites than in the Pacific sites; however, at sediment depths greater than 20 cmbsf, both Pacific and Arctic sites stabilize to ∼500 observed OTUs (Figures 5C,D).

### Taxon-Specific *i*DNA/*t*DNA Ratios

**FIGURE 6:**
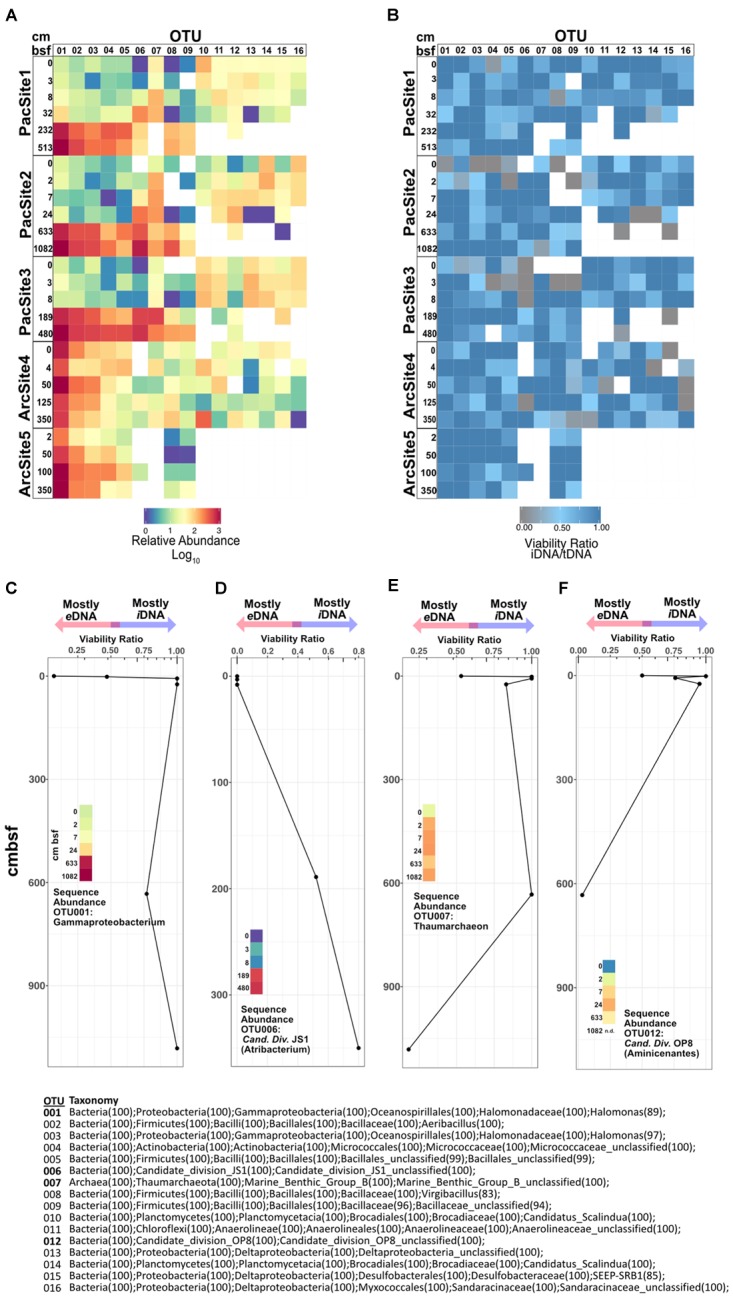
**(A)** Heat map of relative sequence abundance for the 16 most abundant OTUs. **(B)** Heat map depicting viability ratios for the 16 most abundant OTUs in our dataset. Combined viability ratio and sequence abundance for OTUs **(C)** 001, **(D)** 006, **(E)** 007, and **(F)** 012. Color scale for sequence abundance in panels C-F is the same as that in panel **A**. Bottom center list shows taxonomic assignments of all depicted OTUs above.

For highly abundant OTUs, the ratio of sequence relative abundance from *i*DNA to *t*DNA (Figures 6A,B) provides a taxon-specific measure of the proportion of reads from cells with intact membranes. Because an intact membrane is a standard measure of cell viability (Nocker et al., 2006), this ratio also provides a taxon-specific measure of potential viability. Ratios near 1 indicate a negligible contribution of *e*DNA to *t*DNA and thus represent OTUs dominated by potentially viable (membrane-intact) cells. To a first approximation, for any given OTU, its *i*DNA/*t*DNA ratio is lowest at sedimentary depths where its sequence abundance is also low (Figures 6A,B). Depth patterns of *i*DNA/*t*DNA ratios for prevalent OTUs exhibit taxon-specific histories (Figure 6B). Some high abundance OTUs (Figures 6C,D) increase in relative sequence abundance and *i*DNA/*t*DNA ratio with sediment depth. Other OTUs (Figures 6E,F) show low abundance and low *i*DNA/*t*DNA ratios at the seafloor, 0 cmbsf, followed by order-of-magnitude increases in sequence relative abundance and high *i*DNA/*t*DNA ratios with increasing depth.

## Discussion

### Influence of *e*DNA on 16S rRNA Gene Abundance

The impact of *e*DNA on 16S rRNA gene abundance is limited to its intermittent detection in only three of twelve samples collected from sediment depths shallower than 10 cmbsf (two from the Pacific and one from the Arctic, Figure 2). In these three samples, 16S rRNA gene abundances from *t*DNA are inflated by 16S rRNA genes from *e*DNA by 36–50% and 28% in the Pacific and Arctic, respectively. We do not detect higher 16S rRNA gene abundances due to *e*DNA in the other nine triplicate samples. This indicates that *e*DNA disappears rapidly relative to its rate of production. The fourteen samples from depths greater than 10 cmbsf in both Arctic and Pacific sediment do not contain significant amounts of *e*DNA [they exhibit no significant differences in 16S rRNA gene abundance between *t*DNA and *i*DNA samples (Figure 2)]. Our inability to detect significant *e*DNA signals in deeper samples, despite having shown that our method efficiently silences the vast majority of *e*DNA on representative substrates (Supplementary Figure S1), indicates that the quantitative effect of detrital 16S rRNA genes on analyses of subseafloor sedimentary communities is likely minimal. It also indicates that the most cells in the subseafloor communities censused by our survey, whether active or dormant, are likely to retain intact cellular membranes, a prerequisite for chemiosmotic potential and ATP production.

To the extent that detectable *e*DNA occurs in three of our twelve near-seafloor samples, based on 16S rRNA gene abundance as a proxy, we corroborate a previous report of *e*DNA in the first 10 cmbsf of sediment (Dell’Anno and Danovaro, 2005). However, the relatively low increases in *t*DNA caused by *e*DNA in these samples and the absence of detectable *e*DNA in the other 9 samples are far below the contribution of *e*DNA to *t*DNA reported by the previous study (∼90%) (Dell’Anno and Danovaro, 2005). This difference may be attributable to (i) high levels of eukaryotic *e*DNA in marine sediment, not detected by our survey, (ii) inflation of *e*DNA in the previous study by membrane damage and DNA leakage during sample handling and storage (including freezing), and/or (iii) use of 16S rRNA gene abundance, in this study, rather than enzymatic digestions, used in the previous study, to assess DNA pools.

### Influence of *e*DNA on Community Composition

The influence of *e*DNA on phylum-level community composition of the three near-seafloor samples with detectable extracellular 16S rRNA gene inflations is minimal (Figure 3, asterisks). This indicates that the taxonomic diversity and composition of *e*DNA, where present, is similar to that of *i*DNA in these samples. Community mapping using hierarchical binning, based on the highest taxonomic assignment per sequence, also shows similar structure between *i*DNA and *t*DNA communities at greater taxonomic resolution (Figure 4). Two alternative interpretations apply here: (i) given the high degree of similarity between *e*DNA and *i*DNA 16S gene populations, death may occur randomly throughout the community, or (ii) the *e*DNA turns over rapidly in near-seafloor sediment and largely reflects the community of intact cells, meaning that significant differences between *i*DNA and *e*DNA, only rarely observed in this study, may be ephemeral because survivors (represented by *i*DNA) rapidly erase that signal and replace it with their own, as recently suggested for lacustrine sediment and a shallow Baltic Bay core (Vuillemin et al., 2017; Torti et al., 2018). Overall, our observations suggest that in both shallow and deep marine sediment, *e*DNA is a minor component of the *t*DNA pool and does not significantly influence community compositions.

### Influence of *e*DNA on Ecological Metrics

Ordination of 16S rRNA gene data from total and intracellular pools shows little to no influence of *e*DNA on inferred ecological patterns for either the Pacific sites or the Arctic sites in our study (Figures 5A,B). Pacific PCoA plots show that community composition primarily depends on sediment depth, rather than influence of *e*DNA (Figure 5A). PCoA plots of the Arctic samples show site-dependent clustering rather than depth-dependent clustering along axis 1 (Figure 5B). Depth-dependent and site-dependent clustering, observed for the Pacific and Arctic sites, respectively, may be inferred when analyzing the *t*DNA dataset (Figures 5A,B, circles) or the *i*DNA dataset (Figures 5A,B, triangles) independently. This implies that *e*DNA has little or no effect on ordination interpretation of the *t*DNA data. The number of observed OTUs [a critical metric from which community richness, diversity, and evenness (Chao 1, Shannon Entropy and Simpson’s index, respectively) are calculated] from *t*DNA and *i*DNA pools in Arctic and Pacific samples also appears unaffected by *e*DNA (Figures 5C,D). Thus, depth-dependent OTU patterns at the Arctic and Pacific sites follow nearly identical trends for *t*DNA and *i*DNA pools. Together, our ordination and diversity results suggest that *e*DNA has little effect on 16S-based surveys of taxonomic diversity and community composition in subseafloor sediment.

### Taxon-Specific Potential Viability and Source Organisms of *e*DNA Pool Throughout Burial History

Operational taxonomic unit-specific sequence abundance (Figure 6A) coupled with *i*DNA/*t*DNA ratios (Figure 6B) shows, to a first approximation, taxon-specific potential viability in subseafloor sediment (Figures 6C–F). Potential viability is based on the intracellular (potentially viable) vs. extracellular (non-viable) state of DNA in nature. We note that the relative abundance of some OTUs, particularly rare ones, in the PMA-treated library may be affected by the omission of extracellular sequences. However, for highly abundant OTUs, as presented here, this ratio may still be informative. We interpret increases in relative sequence abundance of some OTUs with sediment depth as selective survival rather than net growth because (i) communities in subseafloor sediment are selected from communities present in shallow sediment (Figure 6A) and, overall, (ii) community size decreases with sediment depth (Figure 2). Interestingly, OTUs 001 and 006, Gammaproteobacteria and Atribacteria (Candidate Division JS1) lineages, respectively, have low *i*DNA/*t*DNA ratios near the water-sediment interface and high *i*DNA/*t*DNA ratios at depth (Figures 6C,D). Thus, sequences clustered into these OTUs at depth largely represent *i*DNA or potentially viable cells. Inversely, OTU 007 and 012, lineages within the Thaumarchaeota and the Aminicenantes (Candidate Division OP8), respectively, have high *i*DNA/*t*DNA ratios near the water-sediment interface and low *i*DNA/*t*DNA ratios at depth (Figures 6E,F). Most DNA from these taxa is from potentially viable cells in shallow sediment, but not at depth. Thus, the organisms from which *e*DNA is sourced (microbes that have died in the sediment) change as a function of sediment depth or burial age. This result indicates that the total necromass pool, composed of all extracellular biomolecules resulting from death within the sediment, arises from different lineages at different sediment depths or different times after burial. For example, in the Pacific sites, DNA that clusters into OTUs 010–016 is abundant and largely intracellular in shallow sediment (Figure 6B). However, at depths >10 cmbsf, DNA that clusters into these same OTUs is extracellular and/or degraded beyond detection. Interestingly, the overall effect of taxon-specific *e*DNA influence appears inconsequential at the community level where structure and diversity metrics (Figure 5) are more strongly influenced by environmental factors such as sediment depth.

## Conclusion

Our detection of extracellular DNA in marine sediment confirms that total DNA surveys do not exclusively represent intracellular DNA in this habitat. However, measurable extracellular DNA is present in only 3 of 12 samples from shallow (0–10 cmbsf) sediment. It is absent from all samples of deeper sediment. Although *in situ* mortality presumably releases extracellular DNA, the scarcity of extracellular DNA indicates that it disappears rapidly relative to its production rate, particularly in subseafloor sediment. Even where extracellular DNA is present, extracellular 16S rRNA genes have little influence on overall community composition: they do not impact measures of community richness, relative abundance, phylum-level community composition, or OTU-level ordination in multivariate analyses.

## Author Contributions

GR conceived the study, collected the samples, performed the analyses, interpreted the data, and was the principal author of the manuscript. SJ collected the samples, interpreted the data, and advised GR. RZ performed the analyses and interpreted the data. SD interpreted the data and advised GR. All authors provided editorial comments to the final draft of the manuscript.

## Conflict of Interest Statement

The authors declare that the research was conducted in the absence of any commercial or financial relationships that could be construed as a potential conflict of interest.

## References

[B1] Cangelosi G. A. Meschke J. S. (2014). Dead or alive: molecular assessment of microbial viability. *Appl. Environ. Microbiol.* 80 5884–5891. 10.1128/AEM.01763-14 25038100 PMC4178667

[B2] Caporaso J. G. Lauber C. L. Walters W. A. Berg-Lyons D. Huntley J. Fierer N. (2012). Ultra-high-throughput microbial community analysis on the Illumina HiSeq and MiSeq platforms. *ISME J.* 6 1621–1624. 10.1038/ismej.2012.8 22402401 PMC3400413

[B3] Carini P. Marsden P. Leff J. Morgan E. Strickland M. Fierer N. (2016). Relic DNA is abundant in soils and obscures estimates of soil microbial diveristy. *Nat. Microbiol.* 2:16242. 10.1101/043372 27991881

[B4] Dell’Anno A. Danovaro R. (2005). Extracellular DNA plays a key role in deep-sea ecosystem functioning. *Science* 309:2179. 10.1126/science.1117475 16195451

[B5] D’Hondt S. Inagaki F. Zarikian C. A. Abrams L. J. Dubois N. Engelhardt T. (2015). Presence of oxygen and aerobic communities from sea floor to basement in deep-sea sediments. *Nat. Geosci.* 8 299–304. 10.1038/ngeo2387

[B6] D’Hondt S. Rutherford S. Spivack A. J. (2002). Metabolic activity of subsurface life in deep-sea sediments. *Science* 295:2067. 10.1126/science.1064878 11896277

[B7] D’Hondt S. E. A. (2004). Distributions of microbial acticities in deep subseafloor sediments. *Science* 306:2216. 10.1126/science.1101155 15618510

[B8] Edgar R. C. Haas B. J. Clemente J. C. Quince C. Knight R. (2011). UCHIME improves sensitivity and speed of chimera detection. *Bioinformatics* 27 2194–2200. 10.1093/bioinformatics/btr381 21700674 PMC3150044

[B9] Finkel S. E. Kolter R. (2001). DNA as a nutrient: novel role for bacterial competence gene homologs. *J. Bacteriol.* 183 6288–6293. 10.1128/JB.183.21.6288-6293.2001 11591672 PMC100116

[B10] Foster Z. S. Sharpton T. J. Grunwald N. J. (2017). Metacoder: an R package for visualization and manipulation of community taxonomic diversity data. *PLoS Comput. Biol.* 13:e1005404. 10.1371/journal.pcbi.1005404 28222096 PMC5340466

[B11] Hellevang B. Pedersen R. B. (2005). Magmatic segmentation of the northern knipovich ridge: evidence for high-pressure fractionation at an ultraslow spreading ridge. *Geochem. Geophys. Geosyst.* 6:Q09007 10.1029/2004gc000898

[B12] Holmes R. Minot A. Kérouel R. Hooker B. Peterson B. (1999). A simple and precise method for measuring ammonium in marine and freshwater ecosystems. *Can. J. Fish. Aquat. Sci.* 56 1801–1808. 10.1139/f99-128

[B13] Jørgensen B. B. Marshall I. P. (2016). Slow microbial life in the seabed. *Annu. Rev. Mar. Sci.* 8 311–332. 10.1146/annurev-marine-010814-015535 26209150

[B14] Kallmeyer J. Pockalny R. Adhikari R. R. Smith D. C. D’hondt S. (2012). Global distribution of microbial abundance and biomass in subseafloor sediment. *Proc. Natl. Acad. Sci. U.S.A.* 109 16213–16216. 10.1073/pnas.1203849109 22927371 PMC3479597

[B15] Kozich J. J. Westcott S. L. Baxter N. T. Highlander S. K. Schloss P. D. (2013). Development of a dual-index sequencing strategy and curation pipeline for analyzing amplicon sequence data on the MiSeq Illumina sequencing platform. *Appl. Environ. Microbiol.* 79 5112–5120. 10.1128/AEM.01043-13 23793624 PMC3753973

[B16] Levy-Booth D. J. Campbell R. G. Gulden R. H. Hart M. M. Powell J. R. Klironomos J. N. (2007). Cycling of extracellular DNA in the soil environment. *Soil Biol. Biochem.* 39 2977–2991. 10.1016/j.soilbio.2007.06.020

[B17] Li B. Chen J. (2013). Development of sensitive and specific qPCR assay in conjunction with propidium monoazide for enhanced detection of live *Salmonella* spp. in food. *BMC Microbiol.* 13:273. 10.1186/1471-2180-13-273 24289661 PMC4219372

[B18] Lorenz M. G. Wackernagel W. (1994). Bacterial gene transfer by natural genetic transformation in the environment. *Microbiol. Rev.* 58 563–602. 7968924 10.1128/mr.58.3.563-602.1994 PMC372978

[B19] Lyle M. Koizumi I. Richter C. (1997). ODP Leg 167, Site 104. *Proc. ODP Init. Rep.* 167 175–221.

[B20] Mahnert A. Vaishampayan P. Probst A. J. Auerbach A. Moissl-Eichinger C. Venkateswaran K. (2015). Cleanroom maintenance significantly reduces abundance but not diversity of indoor microbiomes. *PLoS One* 10:e0134848. 10.1371/journal.pone.0134848 26273838 PMC4537314

[B21] McMurdie P. J. Holmes S. (2013). phyloseq: an R package for reproducible interactive analysis and graphics of microbiome census data. *PLoS One* 8:e61217. 10.1371/journal.pone.0061217 23630581 PMC3632530

[B22] Miller D. N. Bryant J. E. Madsen E. L. Ghiorse W. C. (1999). Evaluation and optimization of DNA extraction and purification procedures for soil and sediment samples. *Appl. Environ. Microbiol.* 65 4715–4724. 10543776 10.1128/aem.65.11.4715-4724.1999 PMC91634

[B23] Moissl-Eichinger C. Auerbach A. K. Probst A. J. Mahnert A. Tom L. Piceno Y. (2015). Quo vadis? Microbial profiling revealed strong effects of cleanroom maintenance and routes of contamination in indoor environments. *Sci. Rep.* 5:9156. 10.1038/srep09156 25778463 PMC4361859

[B24] Nocker A. Cheung C. Y. Camper A. K. (2006). Comparison of propidium monoazide with ethidium monoazide for differentiation of live vs. dead bacteria by selective removal of DNA from dead cells. *J. Microbiol. Methods* 67 310–320. 10.1016/j.mimet.2006.04.015 16753236

[B25] Nocker A. Richter-Heitmann T. Montijn R. Schren F. Kort R. (2010). Discrimination between live and dead cells in bacterial communities from environmental water samples analyzed by 454 pyrosequencing. *Int. Microbiol.* 13 59–65. 10.2436/20.1501.01.111 20890840

[B26] Nocker A. Sossa-Fernandez P. Burr M. D. Camper A. K. (2007). Use of propidium monoazide for live/dead distinction in microbial ecology. *Appl. Environ. Microbiol.* 73 5111–5117. 10.1128/AEM.02987-06 17586667 PMC1951001

[B27] Nyström T. (2001). Not quite dead enough: on bacterial life, culturability, senescence, and death. *Arch. Microbiol.* 176 159–164. 10.1007/s002030100314 11511862

[B28] Oksanen J. Blanchet F. G. Kindt R. Legendre P. Minchin P. R. O’hara R. B. (2015). *Vegan: Community Ecology Package. R Package Version 2.2-1*. Available at: http://cran.r-project.org/package=vegan

[B29] Parkes R. J. Webster G. Cragg B. A. Weightman A. J. Newberry C. J. Ferdelman T. G. (2005). Deep sub-seafloor prokaryotes stimulated at interfaces over geological time. *Nature* 436 390–394. 10.1038/nature03796 16034418

[B30] Pietramellara G. Ascher J. Borgogni F. Ceccherini M. T. Guerri G. Nannipieri P. (2008). Extracellular DNA in soil and sediment: fate and ecological relevance. *Biol. Fertil. Soils* 45 219–235. 10.1007/s00374-008-03458

[B31] Postgate J. R. Hunter J. R. (1961). On the survival of frozen bacteria. *J. Gen. Microbiol.* 26 367–378. 10.1099/00221287-26-3367 14488215

[B32] Quast C. Pruesse E. Yilmaz P. Gerken J. Schweer T. Yarza P. (2013). The SILVA ribosomal RNA gene database project: improved data processing and web-based tools. *Nucleic Acids Res.* 41 D590–D596. 10.1093/nar/gks1219 23193283 PMC3531112

[B33] Racine J. S. (2012). RStudio: a platform-independent IDE for R and sweave. *J. Appl. Econom.* 27 167–172. 10.1002/jae.1278

[B34] Ramírez G. A. Graham D. D’hondt S. (2018). Influence of commercial DNA extraction kit choice on prokaryotic community metrics in marine sediment. *Limnol. Oceanogr. Methods* 16 525–536. 10.1002/lom3.10264

[B35] Salter S. Cox M. Turek E. Calus S. Cookson W. O. (2014). Reagent and laboratory contaminantion can critically impact sequence-based microbiome analyses. *BMC Biol.* 12:87. 10.1186/s12915-014-0087-z 25387460 PMC4228153

[B36] Schippers A. Neretin L. N. Kallmeyer J. Ferdelman T. Cragg B. A. Parkes R. J. (2005). Prokaryotic cells of the deep sub-seafloor biospehre identified as living bacteria. *Nature* 433 861–864. 10.1038/nature03302 15729341

[B37] Schloss P. D. Westcott S. L. Ryabin T. Hall J. R. Hartmann M. Hollister E. B. (2009). Introducing mothur: open-source, platform-independent, community-supported software for describing and comparing microbial communities. *Appl. Environ. Microbiol.* 75 7537–7541. 10.1128/AEM.01541-09 19801464 PMC2786419

[B38] Sheik C. S. Reese B. K. Twing K. I. Sylvan J. B. Grim S. L. Schrenk M. O. (2018). Identification and removal of contaminant sequences from ribosomal gene databases: lessons from the census of deep life. *Front. Microbiol.* 9:840. 10.3389/fmicb.2018.00840 29780369 PMC5945997

[B39] Thomas C. M. Nielsen K. M. (2005). Mechanisms of, and barriers to, horizontal gene transfer between bacteria. *Nat. Rev. Microbiol.* 3 711–721. 10.1038/nrmicro1234 16138099

[B40] Torti A. Jorgensen B. B. Lever M. A. (2018). Preservation of microbial DNA in marine sediments: insights from extracellular DNA pools. *Environ. Microbiol.* 10.1111/1462-2920.14401 [Epub ahead of print]. 30198168

[B41] Torti A. Lever M. A. Jørgensen B. B. (2015). Origin, dynamics, and implications of extracellular DNA pools in marine sediments. *Mar. Genomics* 24(Pt 3), 185–196. 10.1016/j.margen.2015.08.007 26452301

[B42] Vaishampayan P. Probst A. J. La Duc M. T. Bargoma E. Benardini J. N. Andersen G. L. (2013). New perspectives on viable microbial communities in low-biomass cleanroom environments. *ISME J.* 7 312–324. 10.1038/ismej.2012.114 23051695 PMC3554398

[B43] Verardo D. J. Froelich P. N. Mcintryre A. (1990). Determination of organic carbon and nitrogen in marine sediments using the Carlo Erba NA-1500 analyzer. *Deep Sea Res.* 37 157–165. 10.1016/0198-0149(90)90034-S

[B44] Vuillemin A. Horn F. Alawi M. Henny C. Wagner D. Crowe S. A. (2017). Preservation and significance of extracellular DNA in ferruginous sediments from lake Towuti, Indonesia. *Front. Microbiol.* 8:1440. 10.3389/fmicb.2017.01440 28798742 PMC5529349

[B45] Walsh E. A. Kirkpatrick J. B. Pockalny R. Sauvage J. Spivack A. J. Murray R. W. (2016). Relationship of bacterial richness to organic degradation rate and sediment age in subseafloor sediment. *Appl. Environ. Microbiol.* 82 4994–4999. 10.1128/AEM.00809-16 27287321 PMC4968545

[B46] Webster G. Newberry C. J. Fry J. C. Weightman A. J. (2003). Assessment of bacterial community structure in the deep sub-seafloor biosphere by 16S rDNA-based techniques: a cautionary tale. *J. Microbiol. Methods* 55 155–164. 10.1016/s0167-7012(03)00140-4 14500007

[B47] Yarza P. Ludwig W. Euzeby J. Amann R. Schleifer K. H. Glockner F. O. (2010). Update of the all-species living tree project based on 16S and 23S rRNA sequence analyses. *Syst. Appl. Microbiol.* 33 291–299. 10.1016/j.syapm.2010.08.001 20817437

