# Supplementary material for: Minimal Influence of Extracellular DNA on Molecular Surveys of Marine Sedimentary Communities
Source: Front Microbiol. 2018 Dec 4;9:2969. doi: 10.3389/fmicb.2018.02969 (PMC6288230; doi:10.3389/fmicb.2018.02969)
Supplement: Supplementary file 1 [file Data_Sheet_1.doc]

*Frontiers in Extreme Microbiology Supplemental Information*

**Minimal influence of extracellular DNA on molecular surveys of marine sedimentary communities**

Gustavo A. Ramírez^1*^, Steffen L. Jørgensen^2^, Rui Zhao^3^, Steven D’Hondt^1^

Author affiliations:

1: University of Rhode Island, Graduate School of Oceanography, Narragansett, RI, USA.

2: University of Bergen, Department of Earth Science, K.G. Jebsen Centre for Deep Sea Research, Bergen, Norway.

3: University of Bergen, Department of Biology, K.G. Jebsen Centre for Deep Sea Research, Bergen, Norway.

*: Corresponding author

**Conflict of interest**:

The authors declare no conflict of interest.

Correspondence:

Dr. Gustavo A. Ramírez

University of Rhode Island

Graduate School of Oceanography

Narragansett Bay Campus

215 South Ferry Road

Narragansett, RI 02882, USA

**Brief summary of DNA spike experiments:**

Autoclaved 0.1 cm^3^ volumes of Pacific and Arctic sediment and 100mg of 1-mm diameter borosilicate glass beads were transferred to sterile 1.5ml translucent centrifuge tubes (in sixtuplicates) and diluted (1:10) with 1x sterile phosphate buffer saline to final volume of 1ml. All tubes were then spiked with 250ng of purified *Vibrio fischeri* chromosomal DNA. This DNA quantity mimics ~10^8^ marine bacterium chromosomoal equivalents per cm^3^ of sediment, assuimng 2.5 fg of DNA per marine bacterial cell (Button and Robertson 2001). All DNA-spiked tubes were vortexed for 10 seconds at maximum speed and placed in a rotator in darkness for 20 mins for homogenization. Subsequently, 3 random tubes from each sixtuplicate subtrate set had PMA (Biotium, Cataloge No. 40013) added to a final concentration of 20uM. Following the same procedure used for environmental samples (see materials and methods in main text), PMA-treated samples (n=3) and untreated controls (n=3) were incubated in darkness at 25°C and 1 atm for 1h with slow mixing *via* hand inversion every 5 mins. Subsequently, PMA-treated samples and untreated controls were exposed on ice to a 500W halogen lamp at a distance of 15 cm for 15 mins with hand mixing *via* inversion every 5 mins. Following DNA extraction, 16S rRNA genes were quantified (n=9) as described in the main text (see experimental procedures). Results are summarized in Figure S1.

**References**
